# Neighborhood Socioeconomic Environment Associated With Aging-Related Immune Phenotypes

**DOI:** 10.1093/geroni/igaf122.893

**Published:** 2025-12-31

**Authors:** Eun Young Choi, Gokul Seshadri, Bharat Thyagarajan, Jennifer Ailshire

**Affiliations:** University of Southern California, Los Angeles, California, United States; University of Minnesota, Minneapolis, Minnesota, United States; University of Minnesota, Minneapolis, Minnesota, United States; University of Southern California, Los Angeles, California, United States

## Abstract

Neighborhood disparities in health and mortality in later life are well-documented, yet their biological underpinnings are not fully understood. Emerging evidence suggests that immune aging, the age-related gradual decline of the immune system, may be a key mechanism driving these disparities. However, little is known about how neighborhood socioeconomic status (NSES) contributes to immune aging. This study examines how neighborhood affluence, deprivation, and specific NSES indicators are associated with immune cell composition, focusing on T-and B-cell subsets. We analyzed individual-level data from a nationally representative sample of adults aged 56+ in the 2016 Health and Retirement Study Venous Blood Study (N = 7,660), merged with neighborhood-level data from the 2014-2018 American Community Survey. We used multilevel regression models, adjusting for census tract clustering and sociodemographic and health-related covariates (e.g., education, comorbidities, CMV seropositivity). Residents of the most affluent neighborhoods had higher CD4+ naïve T cells (45.6% vs. 43.1%, p < 0.001) and lower CD4+ central memory T cells (35.8% vs. 36.8%, p < 0.05) than those in the least affluent neighborhoods. Those in the most deprived neighborhoods had higher IgD- memory B cells (11.9% vs. 11.1%, p < 0.05). Among NSES indicators, neighborhood-level poverty and higher education rates were associated with immune cell profiles less and more capable, respectively, of responding to foreign stressors. Affluent neighborhoods may support immune resilience through lower chronic stress and better access to healthcare, while deprivation may accelerate immune aging potentially through greater antigen exposure or chronic immune activation. Our findings offer important insights into the immunological mechanisms underlying long-standing neighborhood-driven health disparities.

